# 10. THE MOLECULAR MECHANISMS OF SCHIZOPHRENIA FROM GLIAL CELLS PERSPECTIVE

**DOI:** 10.1093/schbul/sby014.033

**Published:** 2018-04-01

**Authors:** Sabina Berretta

**Affiliations:** Harvard Medical School, McLean Hospital

## Abstract

**Overall Abstract:** In the past decade, rapid advances in the field of neuroscience resulted in a dramatic paradigm shift in the way we understand the role of glia in normal brain functions and brain disorder pathology. A growing body of evidence shows that diversified populations of astrocytes, microglia, oligodendrocyte precursors and mature oligodendrocytes play a critical role in the regulation of synaptic functions, blood-brain barrier, immune response regulation, myelination and axonal conduction, and in the synthesis of the extracellular matrix, a key regulator of neural plasticity. Building on this evidence, exciting new findings are beginning to emerge, shedding light on glia abnormalities in schizophrenia and their impact these functions. This symposium aims to discuss and integrate the current state of knowledge on direct evidence for glial abnormalities in schizophrenia and their underlying mechanisms.

Dr. Juliana Nascimiento will present novel findings on the effects of NMDAr antagonists and antipsychotics influence glial cell lines and 3D cultures as neurospheres and cerebral organoids. Results from these studies point to the central role of glycolysis, EIF2 signaling and translational machinery in oligodendrocytes and astrocytes. Dr. Paul Klauser will report on elegant investigations on the implication of developmental redox imbalance inducing oxidative stress leading to impairments of oligodendrocytes, myelin formation and eventually to the disruption of white fibers integrity and conductivity, especially in brain regions where the metabolic demand is high. In patients, alterations of white matter were found to be inversely correlated with blood levels of GSH precursor cysteine and could be prevented by the early administration of the antioxidant N-acetyl cysteine. Dr. Sabina Berretta will discuss recent findings on novel modalities of interaction between glial cells, extracellular matrix and neurons, postulated to affect synaptic structural plasticity and axonal conductance. A growing body of evidence from her group shows disruption of such interactions in schizophrenia, potentially contributing to synaptic pathology and impacting neural connectivity. Dr. Dost Ongur will build on previous work showing abnormal diffusion of neuron-specific metabolite NAA in frontal white matter in patients with chronic schizophrenia in the absence of abnormalities in the diffusion of non-specific metabolites Cr and Cho. State-of-the-art recent studies on first episode psychosis patients and matched healthy controls show that NAA diffusion is normal in first episode patients but Cr and Cho diffusion is abnormal, suggesting that white matter abnormalities in non-neuronal elements in early phases of schizophrenia which are followed by neuronal damage in chronic disease.

